# Dysbiosis of Fecal Microbiota in Tg2576 Mice for Alzheimer’s Disease during Pathological Constipation

**DOI:** 10.3390/ijms232314928

**Published:** 2022-11-29

**Authors:** Ji-Eun Kim, Yu-Jeong Roh, Yun-Ju Choi, Su-Jin Lee, You-Jeong Jin, Hee-Jin Song, A-Yun Seol, Hong-Joo Son, Jin-Tae Hong, Dae-Youn Hwang

**Affiliations:** 1 Department of Biomaterials Science (BK21 FOUR Program), Life and Industry Convergence Research Institute, College of Natural Resources and Life Science, Pusan National University, Miryang 50463, Republic of Korea; 2 Department of Life Science and Environmental Biochemistry, Life and Industry Convergence Research Institute, College of Natural Resources and Life Science, Pusan National University, Miryang 50463, Republic of Korea; 3 College of Pharmacy, Chungbuk National University, Chungju 28644, Republic of Korea; 4 Longevity & Wellbeing Research Center, Laboratory Animals Resources Center, Pusan National University, Miryang 50463, Republic of Korea

**Keywords:** Tg2576 mice, constipation, fecal microbiota, dysbiosis, Alzheimer’s disease

## Abstract

Tg2576 transgenic mice for Alzheimer’s disease (AD) exhibited significant phenotypes for neuropathological constipation, but no research has been conducted on the association of the fecal microbiota with dysbiosis. The correlation between fecal microbiota composition and neuropathological constipation in Tg2576 mice was investigated by examining the profile of fecal microbiota and fecal microbiota transplantation (FMT) in 9–10-month-old Tg2576 mice with the AD phenotypes and constipation. Several constipation phenotypes, including stool parameters, colon length, and histopathological structures, were observed prominently in Tg2576 mice compared to the wild-type (WT) mice. The fecal microbiota of Tg2576 mice showed decreases in Bacteroidetes and increases in the Firmicutes and Proteobacteria populations at the phylum level. The FMT study showed that stool parameters, including weight, water content, and morphology, decreased remarkably in the FMT group transplanted with a fecal suspension of Tg2576 mice (TgFMT) compared to the FMT group transplanted with a fecal suspension of WT mice (WFMT). The distribution of myenteric neurons and the interstitial cells of Cajal (ICC), as well as the enteric nervous system (ENS) function, remained lower in the TgFMT group. These results suggest that the neuropathological constipation phenotypes of Tg2576 mice may be tightly linked to the dysbiosis of the fecal microbiota.

## 1. Introduction

Constipation is tightly linked to many neurological disorders, including Parkinson’s disease (PD), multiple sclerosis (MS), spinal cord injury, and Alzheimer’s disease (AD) [1,2,3,4]. On the other hand, these correlations were focused on mainly in studies related to PD and have rarely been studied in AD, even though constipation has been detected in 4.3% to 17.2% of AD patients [4,5,6]. Recently, some significant constipation phenotypes were first identified in Tg2576 mice showing AD-like phenotypes. The level of the constipation parameters, including stool weight, histological structure, cytological structure, and mucin secretion, were remarkably changed in 11-month-old Tg2576 mice. In addition, the mid colon of the mice showed a decrease in M2 and M3 expression and the downstream signaling pathways of muscarinic acetylcholine receptors (mAChRs) and activated the endoplasmic reticulum (ER) stress proteins and alteration of the ER structure [7]. On the other hand, it is unclear if the constipation phenotypes observed in the Tg2576 mcie were associated with microbiota dysbiosis.

The abnormal composition of intestinal microbiota is closely related to the pathogenesis and some symptoms of chronic constipation. These correlations focused on the fecal microbiota analyses for constipation patients [8,9]. The enhancement in the Bifidobacteria and Clostridium leptum population was first detected in the feces of children with constipation phenotypes [10]. In chronically constipated adults, the population of some microaerophilic or obligate anaerobic bacteria containing Bifidobacterium, Bacteroides spp., and Lactobacillus was decreased significantly, where other groups of bacteria, including *Pseudomonas aeruginosa* and *Campylobacter jejuni*, were increased [11,12,13,14]. An increased population of Bacteroides spp. And Enterobacteriaceae and a decreased population of Bifidobacteria, Clostridium leptum, and Faecalibacterium prausnitzii were observed in patients with constipation-predominant irritable bowel syndrome (IBS-C) [15,16]. A significant decrease or increase in the Prevotella and Firmicutes populations was detected in other constipated patients, even though the Lactobacillus and Bifidobacterium populations remained constant [17]. Moreover, the population of several butyrate-producing genera, including Coprococcus, Faecalibacterium, and Roseburia, was enhanced remarkably in constipated patients [18,19]. Nevertheless, the results obtained from the above studies suggest a discrepancy in the changes in the microbial population between patients.

The changes in the intestinal microbiota profile during constipation were investigated in several constipated animal models manufactured by an injection of several chemical drugs and gene modification techniques. Among these, significant changes in the microbiota abundance were detected in chemical-induced constipation models, which exhibited significant changes in the excretion parameters, gastrointestinal transit, and histological structure of the colon after treatment with three chemicals, including morphine, loperamide (Lop), and phenolphthalein. An abundance of pathogenic bacteria, including Flavobacterium, Enterococcus, Fusobacterium, Sutterella, and Clostridium at the genus level, were enhanced in the morphine-induced constipation model [20]. In the mice with constipation induced by phenolphthalein, the population of Firmicutes and Actinobacteria declined, and Bacteroidetes were enhanced remarkably [21]. The Lop-induced constipation model showed a decrease in the Bacteroidetes population and an increase in Firmicutes and Proteobacteria at the phylum level [22,23]. The Lactobacillaceae (five days) and Deslfovibrionaceae (12 days) populations were found in other constipated models injected with Lop [24]. On the other hand, no changes in the intestinal microbiota were observed in the activated carbon-induced constipation model [25]. Moreover, a similar alteration pattern in the intestinal microbiota population was examined in a few genetically engineered mouse (GEM) models with constipation phenotypes. Complement 3 (C3) knockout (KO) mice with constipation phenotypes showed significant decreases in several bacteria populations, including Anaerocolumna, Caecibacterium, Christensenella, Kineothrix, and Oscillibacter, as well as remarkable increases in the Bacteroides, Culturomica, Eubacterium, Muribaculum, Prevotellamassilia, Reuthenibacterium, and Prevotella populations [26,27]. On the other hand, these changes in the intestinal microbiota were not analyzed in a neuropathological constipation model with AD-like phenotypes.

This study compares the fecal microbiota profile in 9–10-month-old Tg2576 mice with neuropathological constipation. The role of their fecal dysbiosis on the defecation delay of Tg2576 mice is verified using an FMT study in AiDM-ICR mice.

## 2. Results

### 2.1. Confirmation of the Constipation Phenotypes in Tg2576 Mice

**Figure 1:**
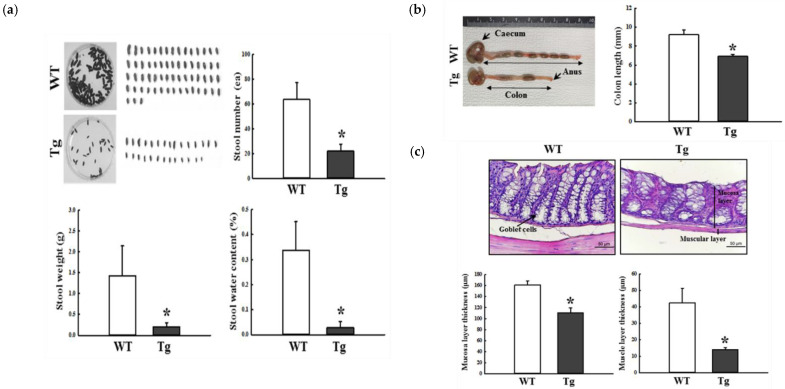
Stool parameters, colon length, and histopathological structures of the mid colon in Tg2576 mice. (**a**) Stool parameters. After collecting and taking digital camera images of the stools, the changes in the number, weight, and water contents of the stools were measured, as described in Materials and Methods. Stools were collected from three to five mice per group, and each parameter was assayed in duplicate. (**b**) Colon length. After collecting the colon, the total length was measured from the caecum to the anus. Five to six mice per group were used to prepare the colon, and the colon length was measured in duplicate. (**c**) Histological analysis. After staining with H&E solution, histological features were observed at 400× magnification using an optical microscope. Alteration of their parameters was determined using the Leica Application Suite. H&E-stained slides were prepared from three to five mice per group, and the histopathological parameters were measured in duplicate in each tissue section. The data are reported as the mean ± SD values. *, *p* < 0.05 compared to the WT group. Abbreviations: WT, wild-type; Tg, Tg2576 mice; H&E, hematoxylin and eosin.

The phenotypes for the defecation delay of Tg2576 mice were detected using the method described in our previous study to confirm the successful induction of constipation [7]. The number, weight, and water content of the stools were significantly lower in the Tg2576 mice than in the WT group, but the rate of the decrease varied. The shape of the stool from the Tg2576 mice changed to an irregular shape, while the regular form was maintained in the WT mice (Figure 1a). A similar decrease in the colon length of the Tg2576 mice was detected (Figure 1b). The abnormal histopathological structure of the mid colon in the Tg2576 mice was observed on the crypt of Lieberkuhn, mucosal layer, and muscle layer. In particular, the thicknesses of the mucosal and muscle layers were significantly lower in the Tg2576 mice than in the WT mice (Figure 1c). These results suggest that the constipation phenotypes could be detected successfully in 9–10-month-old Tg2576 mice, allowing an investigation of the dysbiosis of fecal microbiota.

### 2.2. Alteration of the Profile of the Fecal Microbiota in Tg2576 Mice with the Constipation Phenotypes

**Figure 2:**
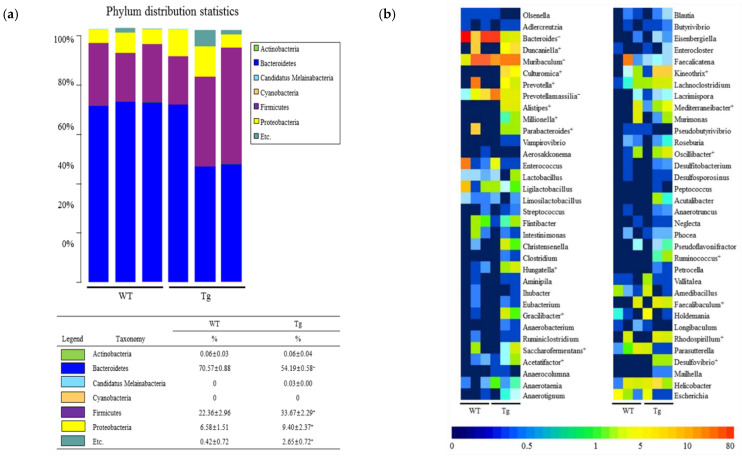
Profile of fecal microbiota. (**a**) Heat map showing the fecal microbiota distribution at the phylum level. Different colors in the heat map indicate the relative abundance of each phylum. (**b**) Heat map showing fecal microbiota distribution at the genus level. Different colors in the heat map indicate the relative abundance of each genus. The data are reported as the mean ± SD values. “−” represents a decrease compared to the WT mice; “+” represents an increase compared to the WT mice. Abbreviations: WT, wild-type; Tg, Tg2576 mice.

**Figure 3:**
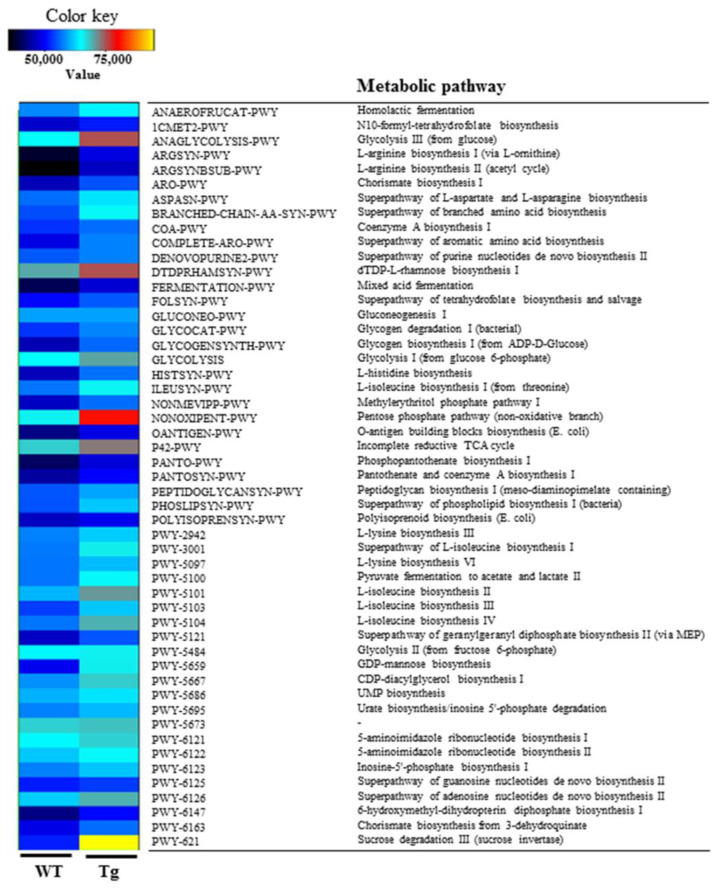
Heat map of the predicted metabolic pathways for the fecal microbiota in Tg2576 mice. This pathway was obtained using the PICRUSt2 (v.2.3.0) software as described in Materials and Methods. Abbreviations: WT, wild-type; Tg, Tg2576 mice.

**Figure 4:**
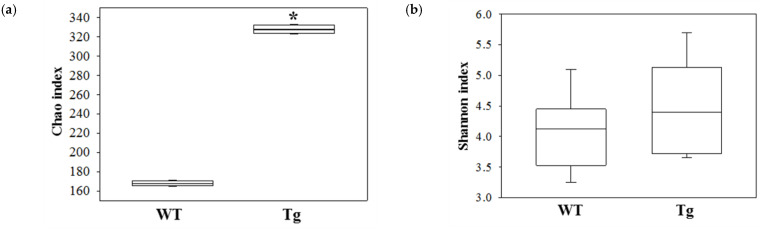
Comparison of (**a**) Chao index and (**b**) Shannon diversity index of the fecal microbiota in Tg2576 mice. The data are reported as mean ± SD values. *, *p* < 0.05 compared to the WT mice. Abbreviations: WT, wild-type; Tg, Tg2576 mice.

The overall microbial composition was analyzed in fecal samples of WT and Tg2576 mice to determine if the defecation delay of the Tg2576 mice affects the fecal microbiota profile. A significant alteration between the WT and Tg2576 mice was detected in three microbial phyla. The population of Bacteroidetes decreased by 20% in the Tg2576 mice compared to the WT mice. In contrast, the Firmicutes and Proteobacteria population increased significantly, with 50.58% and 42.7% in the same group (Figure 2a,b). In addition, genus-level analyses detected 19 populations with significant alterations. The population of three genera, including *Bacteroides*, *Prevotellamassilia*, and *Muribaculum*, were increased significantly in the fecal samples of Tg2576 mice compared to WT mice. Among them, *Bacteroides* (94%) showed the largest increase at the genus level, followed in order by *Prevotellamassilia* (44%) and *Muribaculum* (29%) (Figure 2b). Furthermore, the proportions of various microbial genera, including Duncaniella, Culturomica, Prevotella, Alistipes, Mediterraneibacter, Rhodospirillum, Faecalibaculum, Oscillibacter, Gracilibacter, Parabacteroides, Saccharofermentans, Desulfovibrio, Millionella, Ruminococcus, Hungatella, Kineothrix, and Acetatifactor, were lower in the Tg2576 mice than the WT mice. The largest decrease in the microbial genera in the Tg2576 mice was observed for Duncaniella (588.9-fold), followed in order by Culturomica (584.9-fold), Prevotella (418.4-fold), Alistipes (350.3-fold), Mediterraneibacter (316.9-fold), Rhodospirillum (308.6-fold), Faecalibaculum (281.5-fold), Oscillibacter (222.6-fold), Gracilibacter (196.7-fold), Parabacteroides (140.1-fold), Saccharofermentans (134.3-fold), Desulfovibrio (131.2-fold), Millionella (103.5-fold), Ruminococcus (94.8-fold), Hungatella (18.4-fold), Kineothrix (9.9-fold), and Acetatifactor (6.3-fold) (Figure 2b). The PICRUSt2 data and analysis revealed 49 predicted metabolic pathways for the fecal microbiota of Tg2576 mice (Figure 3). On the other hand, the Chao index and Shannon index for species richness and diversity were increased in Tg2576 mice compared to WT mice, even though statistical significance was observed only in the Chao index (Figure 4). Therefore, the defecation delay of Tg2576 mice may be closely linked to the dysbiosis of the fecal microbiota in Tg2576 mice at the phylum and genus levels.

### 2.3. Effects of TgFMT on Fecal Parameters of AiDM-ICR Mice

**Figure 5:**
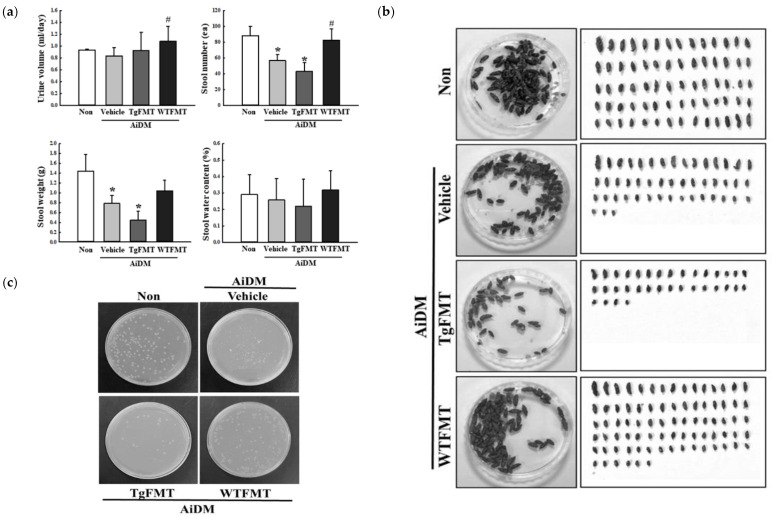
Excretion parameters and growth of feces-derived bacteria in AiDM+TgFMT mice. (**a**) Excretion parameters. The urine volume, stool number, stool weight, and stool water contents were measured, as described in Materials and Methods. (**b**) Stool morphological characteristics. After collecting stools from a metabolic cage, they were arranged according to size, and digital camera images were taken immediately. (**c**) Cultivation of feces-derived bacteria. The growth pattern of bacteria colonies was observed and analyzed on a view box. The stool collection was prepared from three to five mice per group, and each stool parameter was analyzed in duplicate. The data are reported as the mean ± SD values. *, *p* < 0.05 compared to the Non-treated group. #, *p* < 0.05 compared to the AiDM+Vehicle group. Abbreviations: WT, wild type; Tg, Tg2576 mice; FMT, fecal microbiota transplantation; TgFMT, Tg2576 mice FMT; WFMT, wild-type mice FMT; AiDM, antibiotics-induced depletion of microbiota.

An FMT study was performed in AiDM-ICR mice for 15 days to determine if the dysbiosis of fecal microbiota in Tg2576 mice with neuropathological constipation can induce defecation delay. The number of stools was significantly lower in the AiDM+TgFMT group than in the AiDM+Vehicle and AiDM+WTFMT group. On the other hand, the water contents of stools and the urine volume were similar in the AiDM+TgFMT group and AiDM+WTFMT group (Figure 5a). A similar change was detected in the morphological properties of the stools. Small, thin, and hard stools accounted for a large portion of the total stools in the AiDM+TgFMT group (Figure 5b). In addition, the growth patterns of the fecal-derived bacteria in nutrient agar plates reflected the changes in stool number. The total number of bacterial colonies on the agar plates was lower in the AiDM+TgFMT group than in the AiDM+Vehicle and AiDM+WTFMT groups (Figure 5c). Hence, the dysbiosis of fecal microbiota in Tg2576 mice may be caused by the defecation delay effects in AiDM-ICR mice.

### 2.4. Effects of TgFMT on the Colon Length and Gastrointestinal (GI) Motility in AiDM-ICR Mice

**Figure 6:**
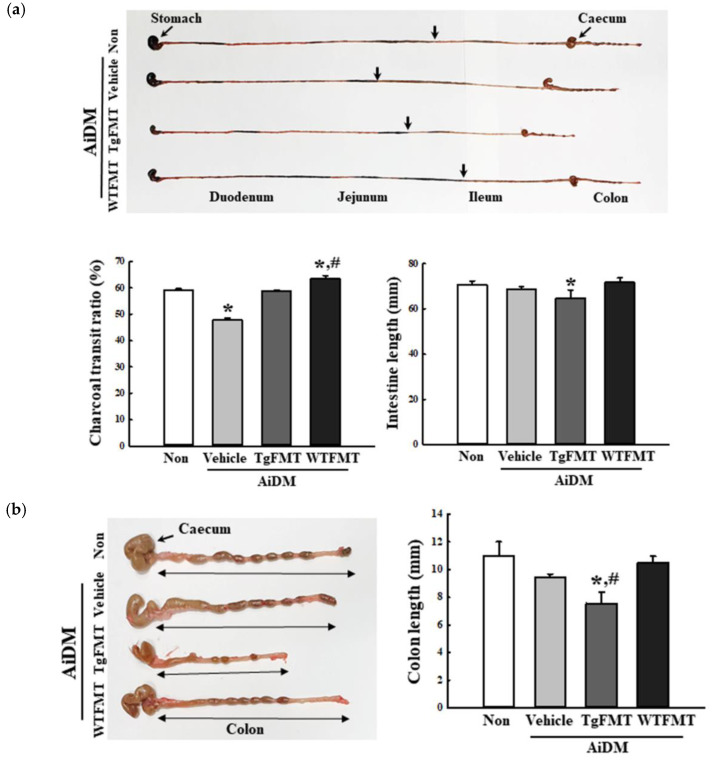
GI transit ratio and colon length in the AiDM+TgFMT group. (**a**) GI transit ratio of the charcoal meal and the length of the colon. After collecting the GI track from AiDM+TgFMT and AiDM+WTFMT groups treated with charcoal meal, the GI length and the distance traveled by the charcoal meal were measured using a ruler. The arrows indicate the position of the charcoal meal. (**b**) Colon length. After harvesting the colon from the total GI track, the colon length was measured similarly. The treatment of charcoal meal powder was prepared from four to six mice per group, and the charcoal meal transit distance and colon length were measured in duplicate. The data are reported as the mean ± SD values. *, *p* < 0.05 compared to the Non-treated group. #, *p* < 0.05 compared to the AiDM+Vehicle group. Abbreviations: WT, wild-type; Tg, Tg2576 mice; FMT, fecal microbiota transplantation; TgFMT, Tg2576 mice FMT; WFMT, wild-type mice FMT; AiDM, antibiotics-induced depletion of microbiota.

The changes in the colon length and GI motility were measured in AiDM+WTFMT and AiDM+TgFMT mice to determine if the TgFMT-induced defecation delay effects were accompanied by alterations in colon length and GI motility. The transit ratio of the charcoal meal was 20% lower in the AiDM+Vehicle-treated group than in the Non-treated group. On the other hand, these levels were significantly higher in the AiDM+TgFMT and AiDM+WTFMT groups, but their increase rate was greater in the AiDM+WTFMT group than the AiDM+TgFMT group (Figure 6a). A similar pattern was observed in the colon length. This level was remarkably lower in the AiDM+TgFMA group than in the AiDM+Vehicle group. Nevertheless, the AiDM+WTFMT group was similar to the AiDM+Vehicle group (Figure 6b). These results suggest that TgFMT-induced defecation delay effects help regulate the colon length and gastrointestinal motility in AiDM-ICR mice.

### 2.5. Effects of TgFMT on the Histopathological Structure of the Mid Colon in AiDM-ICR Mice

**Figure 7:**
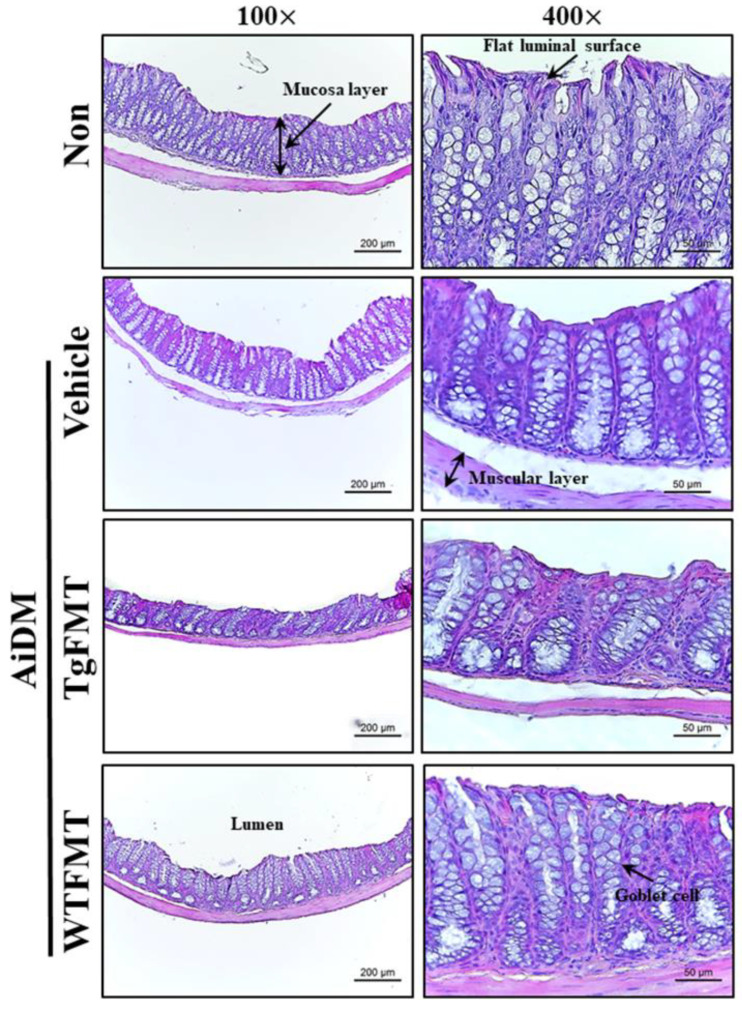
Histopathological structures of the mid colon in the AiDM+TgFMT mice. After staining the sections of the mid colon with H&E solution, histopathological structures were observed and characterized at 100× (**left** column) and 400× (**right** column) using an optical microscope. H&E-stained slides of the mid colon were prepared from four to six mice per group, and each parameter in their structure was measured in duplicate. Abbreviations: WT, wild-type; Tg, Tg2576 mice; FMT, fecal microbiota transplantation; TgFMT, Tg2576 mice FMT; WFMT, wild-type mice FMT; AiDM, antibiotics-induced depletion of microbiota.

Next experiment examined whether alterations in the histological structure of the mid colon were accompanied by TgFMT-induced defecation delay effects in the AiDM-ICR mice. The histological parameters indicating constipation phenotypes were analyzed in the H&E-stained mid colons of the AiDM+TgFMT and AiDM+WTFMT mice. The thicknesses of the mucosa and muscular layer were significantly lower in the AiDM+TgFMT group than those of the AiDM+Vehicle or Non-treated groups, while these levels were constantly maintained in the AiDM+WTFMT group (Figure 7). In addition, AiDM+TgFMT mice exhibited the typical histological structure of the mid colon observed in constipation models. They included an irregular shape and unequal distribution of crypts of Lieberkuhn and goblet cells with various sizes compared with the AiDM+WTFMT group. On the other hand, a relatively normal structure was maintained in the mid colon section of the AiDM+WTFMT group (Figure 7). These results suggest that the TgFMT-induced defecation delay effects may contribute to structural abnormalities in the mid colon of AiDM-ICR mice.

### 2.6. Effects of TgFMT on the Expression of Water Channels in the Mid Colon of AiDM-ICR Mice

**Figure 8:**
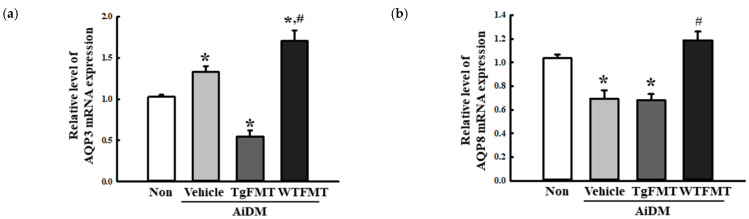
Expression levels of the AQPs in AiDM+TgFMT mice. The relative levels of AQP3 (**a**) and AQP8 (**b**) transcripts of the total mRNA from mid-colons were measured by RT-qPCR analyses using specific primers. The mRNA levels of the two genes were represented as the relative level of the intensity of β-actin. The total RNA was prepared from three to five mice per group, and RT-qPCR analyses were performed in duplicate. The data are reported as the mean ± SD values. *, *p* < 0.05 compared to the Non-treated group. #, *p* < 0.05 compared to the AiDM+Vehicle group. Abbreviation; WT, wild type; Tg, Tg2576 mice; FMT, Fecal microbiota transplantation; TgFMT, Tg2576 mice FMT; WFMT, Wild type mice FMT; AiDM, Antibiotics-induced depletion of microbiota.

The changes in the expression level of water channels were analyzed in the mid colon of AiDM+TgFMT mice to determine if the TgFMT-induced defecation delay effects are accompanied by alterations in the regulation of water secretion. The levels of aquaporin-3 (AQP3) and APQ8 expression in the AiDM+TgFMT group were remarkably lower at 47% and 30% than in the Non-treated group, even though the decrease rate was greater in AQP3 mRNA than AQP8 mRNA. On the other hand, these levels were higher in the AiDM+WTFMT group (Figure 8a,b). These results show that the TgFMT-induced defecation delay effects are associated with a decreased ability to secrete water through the alternative expression of the water channel protein in the mid colon of AiDM-ICR mice.

### 2.7. Effects of TgFMT on the Distributions of Neuronal Cells and ICC in the Mid Colon of AiDM-ICR Mice

**Figure 9:**
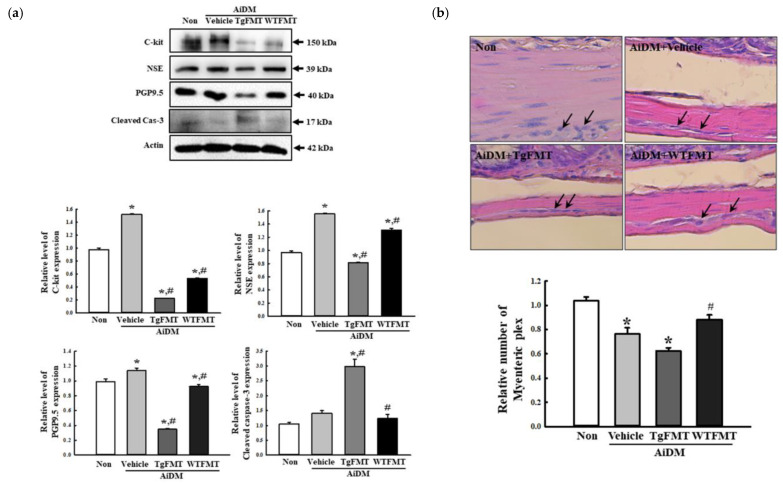
Levels of markers for neuronal cells and ICC in AiDM+TgFMT mice. (**a**) Expression levels of C-kit, NSE, PGP9.5, and Cleaved Cas-3 proteins. The expression of the four proteins in the mid colon was measured with western blot analysis using a specific antibody. The expression level of each protein were represented as the relative level of the β-actin intensity. (**b**) Histological distribution of myenteric plexus. H&E-stained sections of the mid colon from AiDM+WTFMT mice and AiDM+TgFMT mice were observed at 400× magnification using a light microscope. Black arrow indicated myenteric plexus. H&E-stained slides of the mid colon were prepared from four to six mice per group, and each parameter in their structure was measured in duplicate. The data are reported as the mean ± SD values. *, *p* < 0.05 compared to the Non-treated group. #, *p* < 0.05 compared to the AiDM+Vehicle group. Abbreviations: WT, wild-type; Tg, Tg2576 mice; FMT, fecal microbiota transplantation; TgFMT, Tg2576 mice FMT; WFMT, wild-type mice FMT; AiDM, antibiotics-induced depletion of microbiota.

The changes in the expression levels of the specific markers, including C-kit, NSE, and PGP9.5 proteins, were measured in the mid colon of AiDM ICR mice to determine if the TgFMT-induced defecation delay effects are accompanied by alterations in the distribution of neuronal cells and ICC. The expression levels of these proteins were significantly lower in the AiDM+TgFMT mice than in the AiDM+Vehicle mice, while AiDM+WTFMT mice showed a constant level. Among them, the greatest decrease was detected in the expression level of PGP9.5 proteins in AiDM+TgFMT mice (Figure 9a). In addition, the reduction in the expression level of PGP9.5 proteins were reflected in the increase in the Cleaved Cas-3 level (Figure 9a). Furthermore, the relative number of myenteric plexus was significantly lower in the AiDM+TgFMT mice than in the AiDM+Vehicle mice (Figure 9b). These results suggest that the TgFMT-induced defecation delay effects are associated with a decrease in the distribution of neuronal cells and ICC in the mid colon of AiDM ICR mice.

### 2.8. Effects of TgFMT on the Excitatory Function of the ENS in the Mid Colon of AiDM-ICR Mice

**Figure 10:**
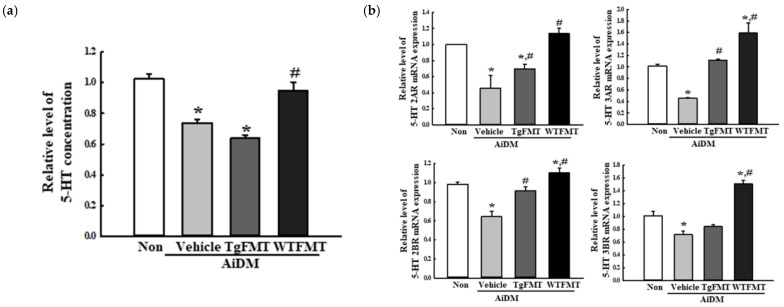
Levels of 5-HT and its receptor mRNA in AiDM+TgFMT mice. (**a**) Level of 5-HT for the excitatory function. The concentration of 5-HT was measured in the mid colon homogenate by an Enzyme-Linked Immunosorbent Assay. The detection range of this kit is 1.5–250 ng/mL of 5-HT. (**b**) Levels of 5-HT 2AR, 2BR, 3AR, and 3BR mRNA. The levels of the four transcripts in the total mRNA of the mid colons were measured by RT-qPCR analysis using the specific primers. The mRNA levels of the two genes were represented as the relative level of the intensity of β-actin. The tissue homogenate and total RNAs were prepared from three to five mice per group, and western blot, ELISA, and RT-qPCR analyses were performed in duplicate. The data are reported as the mean ± SD values. *, *p* < 0.05 compared to the Non-treated group. #, *p* < 0.05 compared with the AiDM+Vehicle treated group. Abbreviations: WT, wild-type; KO, knock out; FMT, fecal microbiota transplantation; KFMT, knockout mice FMT; WFMT, wild-type mice FMT; AiDM, antibiotics-induced depletion of microbiota; AiDM-WT, AiDM wild-type mice; AiDM-KO, AiDM knockout mice.

**Figure 11:**
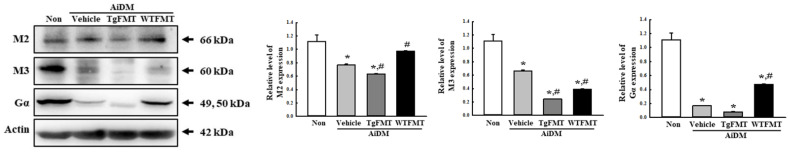
Levels of mAChRs expression in AiDM+TgFMT mice. mAChR M2, M3, and Gα expression. The expression levels of mAChR M2, M3, and Gα were measured by western blot analysis using the specific primary antibodies and HRP-labeled anti-rabbit IgG antibody. The tissue homogenate was prepared from three to five mice per group, and western blot analyses were performed in duplicate. The data are reported as the mean ± SD values. *, *p* < 0.05 compared to the Non-treated group. #, *p* < 0.05 compared to the AiDM+Vehicle-treated group. Abbreviations: WT, wild-type; KO, knock out; FMT, fecal microbiota transplantation; KFMT, knockout mice FMT; WFMT, wild-type mice FMT; AiDM, antibiotics-induced depletion of microbiota; AiDM-WT, AiDM wild-type mice; AiDM-KO, AiDM knockout mice.

Finally, this study investigated whether TgFMT-induced defecation delay effects are accompanied by alterations in the excitatory function of the ENS. To achieve this, the change in the regulatory factors for the 5-HT and ACh function were analyzed in the mid colon of AiDM-ICR mice with constipation. The concentration of 5-HT was significantly lower in the AiDM+TgFMT mice than in the AiDM+Vehicle mice, but this level was maintained in AiDM+WTFMT mice (Figure 10a). In addition, a similar decrease was observed in the mRNA levels of four 5-HT receptors. The transcription levels of 2AR, 2BR, 3AR, and 3BR were higher in the AiDM+TgFMT and AiDM+WTFMT groups than the AiDM+Vehicle group, but the increase rate was greater in the AiDM+WTFMT group than the AiDM+TgFMT group (Figure 10b). Furthermore, a similar decrease in mAChR expression was observed. The levels of mAChR M2, M3, and Gα proteins were significantly lower in the mid colon of the AiDM+TgFMT mice than in the AiDM+Vehicle mice (Figure 11). Overall, the above results show that TgFMT-induced defecation delay effects may be tightly linked to the decreased dysregulation of the excitatory function of the ENS in the mid colon of AiDM-ICR mice.

## 3. Discussion

The changes in the microbiota composition of the intestines are closely related to a dysregulation of gastrointestinal mobility, maturation of gut-associated lymphoid tissue, mucosal barrier function, and repair of tissue damage [17,28,29]. Hence, the microbiota profile was analyzed under various constipation conditions to determine the correlation between the constipation phenotypes and microbial composition. This study investigated the role of the fecal microbiota in Tg2576 mice with neuropathological constipation through microbiota composition analysis and FMT experiments. The results show that significant changes in the fecal microbiota profile could be detected in Tg2576 mice with neuropathological constipation. In addition, the FMT study showed that fecal microbiota derived from constipated Tg2576 mice caused defecation delay in AiDM-ICR mice.

The Tg2576 mice used in this study were first manufactured by microinjecting the human amyloid precursor proteins (APP) 695 gene containing the Swedish-type mutation (K670N, M671L) under the control of a hamster prion protein into B6SJLF2 fertilized eggs [30]. They exhibited severe AD phenotypes, such as accumulation of amyloid β (Aβ)-42, formation of extracellular amyloid plaques, and behavioral dysfunction, as major symptoms [29]. This model was accompanied by several other clinical disease symptoms, including obesity, aortic atherosclerosis, insulin resistance neuroinflammation, and a loss of noradrenergic neurons [31,32,33,34]. A recent study reported that chronic constipation is accompanied by other symptoms in this animal model. This study investigated the role of the intestinal microbiota in Tg2576 mice as a follow-up study on constipation symptoms. These findings provide additional scientific evidence that the neuropathological constipation of Tg2576 mice may be associated with significant changes in the profile of fecal microbiota. 

In the present study, significant changes at the phylum level were detected in only three populations of fecal microbiota from Tg2576 mice. Firmicutes and Proteobacteria were increased in Tg2576 mice, while Bacteroides decreased in the same group, as shown in Figure 2a. Similar alterations in the population of Bacteroidetes, Firmicutes, and Proteobacteria were observed in the Lop-induced constipation model, despite some differences in a few phylum populations between the control and Lop-treated groups [22,23,24]. On the other hand, an opposite alteration pattern was observed in phenolphthalein-induced constipation models and the C3 KO model with constipation phenotypes. They revealed increases in Bacteroidetes and decreases in Firmicutes, but there were differences in their rates of change [21,27]. These results suggest that Bacteroidetes and Firmicutes are important indicators of the constipation phenotypes despite the differences between model animals. Furthermore, more differences were observed in the analysis at the genus level. These results, which show an increase in three genera and a decrease in 16 genera, are not similar to the other three models, including morphine-, phenolphthalein- and loperamide (Lop)-induced models [20,21,24]. On the other hand, some genera with similar alterations were observed in C3 KO mice. Significant increases in Bacteroidetes, Muribaculum, and Prevotellamassilia, and a decrease in Kineothrix and Oscillibacter, were commonly detected in Tg and C3 KO mice during the development of constipation phenotypes [27]. The differences between the present results and previous studies were attributed to differences in the characteristics of the treated chemicals, breeding conditions, and background strain of animals used in the experiments. Hence, further studies are needed to address this issue.

The results of fecal microbiota detected in this study cannot be related entirely to the constipation phenotypes of Tg2576 mice because these mice also have the AD phenotypes [7]. The mice at 9–10 months of age exhibited significant constipation phenotypes, including decreased stool weight and number, abnormality of histological structure, and suppression of mucin secretion and AD-like phenotypes [7]. Thus far, the changes in the gut microbiota composition cause the pathogenesis of AD based on microbiota composition analysis for AD animal models and patients [35]. A significant alteration was detected in six phyla, including Actinobacteria, Bacteroidetes, Firmicutes, Proteobacteria, Tenericutes, and Verrucomicrobia. On the other hand, the changing pattern of each phylum varied in different animal models, such as APP/presenilin (PS1), five AD-linked mutations (5X FAD), Tg2576, and P301L mice [35,36]. This study identified changes in only Bacteroidetes, Firmicutes, and Proteobacteria phyla in the fecal samples of Tg2576 mice with constipation without any changes in the other phyla. These and previous results suggest only three phyla are associated with the phenotypes of constipation and AD simultaneously. Therefore, these groups can be used as targets for diagnosing and treating constipation and AD.

Despite the above similarity of gut microbiota at the phylum level in constipation and AD, very few genera were detected in the two disease conditions simultaneously. Some AD models showed a change in the microbiota, including Bacteroides, Odoribacter, Alloprevotella, Allobaculum, Roseburia, Lactobacillus, Ruminococcus, Desulfovibrio, and Helicobacter, during the development of pathology phenotypes [35,36]. On the other hand, the present study detected different genera representing significant changes, as shown in Figure 2c. Only Bacteroides were commonly detected in the constipation and AD models. These bacteria were distributed in the digestive tract as anaerobic, gram-negative, and non-spore-forming bacteria [37]. Furthermore, most Bacteroides provide some benefits to their host by excluding potential pathogens from colonizing the gut, even though some species of this group play a role of opportunistic human pathogens [38]. Therefore, these results provide scientific evidence that Bacteroides at the genus level can play an essential role in chronic constipation and AD. Nevertheless, further research is needed on how Bacteroides work in the pathogenesis of both diseases.

## 4. Materials and Methods

### 4.1. Management of Animal Study

The protocol for the animal experiment was reviewed and approved by the Pusan National University Institutional Animal Care and Use Committee (PNU-IACUC; Approval Number PNU-2021-2879). WT and Tg2576 mice were bred at the PNU-Laboratory Animal Resources Center, which is accredited by AAALAC International (Number: 001525) and the Korea Food and Drug Administration (FDA, Number: 000231). WT and Tg2576 mice were provided *ad libitum* access to a standard irradiated chow diet (Samtako BioKorea Co., Osan, Republic of Korea) and drinking water. All adult mice for this study were bred under a specific pathogen-free state (SPF) with a relative humidity of 50 ± 10%, temperature of 22 ± 2 °C, and a strict light cycle (lights on at 06:00 h and off at 18:00 h).

### 4.2. Experimental Design of the Tg2576 Mice

The Tg2576 mice were first produced at the University of Minnesota by a microinjection of the APP695sw gene into zygotes of B6SJLF2 mice. A high concentration of Aβ peptides, amyloid plaques, and memory deficits were detected in these mice at 9–10 months of age [30]. Seven-week-old Tg2576 mice and B6SJL were first provided from Samtaco Biokorea Co., Taconic branch, Korea. These mice were bred at the PNU-Laboratory Animal Resources Center until the age acceptable for the experiment.

When Tg2576 mice (*n* = 12) and WT mice (*n* = 12) reached 9–10 months of age, seven to nine mice were used for the stool parameter and colon length analysis, and three mice were used for the microbiota experiment. To achieve these, each mouse of the subset group was placed in a single metabolic cage for 12 h. Briefly, all stools of each mouse were collected independently from each cage and subjected to stool parameter and microbiota analyses. Subsequently, all mice of the subset groups were sacrificed immediately with CO_2_ gas after fasting for 18 h. Finally, the tissue samples were collected from all mice and stored in 10% formalin solution and Eppendorf tubes at −70 °C until assayed.

### 4.3. Excretion Parameters Analyses

Stools and urine samples for excretion parameters analyses were collected from each mouse of the subset group using individual metabolic cages (Daejong Ltd., Seoul, Republic of Korea) for 24 h. Subsequently, the total number of stools was directly counted twice per mouse, and the weight was measured twice per sample using an electric balance (Mettler Toledo, Columbus, OH, USA) to ensure statistical accuracy. In addition, the moisture content of stools was calculated using the following calculation method:

Moisture content of stools = (A − B)/A × 100

where A is the weight of fresh stools, and B is the weight of dry stools after treatment at 60 °C for 24 h. Furthermore, the urine volume was measured twice per sample using a cylinder after harvesting from the individual metabolic cages to avoid contamination.

### 4.4. Measurement of GI Transit Ratio and Colon Length

The GI transit ratio, and total GI track and colon length were measured using the method described elsewhere [39,40]. Briefly, the mice fasted for 18 h and were administered 0.3 mL of a charcoal meal (3% suspension of activated charcoal in 0.5% aqueous methylcellulose) (Sigma-Aldrich Co., St. Louis, MO, USA). After 30 min of administration, they were euthanized with CO_2_, and the GI track was collected from the abdominal cavity. The GI transit ratio was calculated using the following calculation method:

GI transit ratio (%) = [(total GI length − transit distance of charcoal meal)/total GI length)] × 100

In addition, the GI length from the stomach to the anus and the colon length from the caecum to the anus were measured in duplicate using a ruler.

### 4.5. Histopathological Analysis of Mid Colon

The histopathological structure of the mid colon was analyzed using the method described elsewhere [41]. After collecting the mid colon samples, they were fixed in 10% formalin for 48 h, embedded in paraffin wax, and then sectioned into 4 μm thick slices. These mid colon sections were stained with hematoxylin and eosin (H&E, Sigma-Aldrich Co.) and permanently preserved with mounting medium (Carl Roth GmbH+Co. KG, Karlsruhe, Germany). The histopathological features in the H&E-stained sections were observed by optical microscopy, after which the mucosal and muscular layer thicknesses were measured using the Leica Application Suite (Leica Microsystems, Wetzlar, Switzerland).

### 4.6. Analysis of Fecal Microbiota

The fecal microbiota were analyzed as described elsewhere [27]. The total DNA of fecal microbiota was extracted from the fresh fecal samples (1 g) from a single mouse (*n* = 3 in the subset group) using a DNeasy Power Soil Kit (Qiagen, Hilden, Germany). Sequencing libraries were prepared based on the Illumina 16S Metagenomic Sequencing Library protocols. A polymerase chain reaction (PCR) was performed under 1× reaction buffer, 1 nM of deoxynucleotide (dNTP) mix, 500 nM each of the universal F/R PCR primer, and 2.5 U of Herculase II fusion DNA polymerase (Agilent Technologies, Santa Clara, CA, USA). The 1st PCR and the 2nd PCR were performed with Universal primer and NexteraXT Indexed primer using the method described elsewhere [27] (Supplement Table S1). After the 2nd PCR, the final purified PCR product was quantified based on the qPCR Quantification Protocol Guide (KAPA Library Quantification kits for IlluminaSequencing platforms) and qualified using the TapeStation D1000 ScreenTape (Agilent Technologies, Waldbronn, Germany). The paired-end (2 × 300 bp) sequencing was performed using the Macrogen unit on the MiSeq™ platform (Illumina, San Diego, CA, USA). MiSeq raw data were curated using the Fastp program [42] and assigned to Operational Taxonomic Units (OTUs) using the Cluster Database at High Identity with Tolerance (CD-HIT-OUT) [43]. A representative sequence of each OTU was aligned according to BLAST^+^ (V2.9.0) [44] in the Reference DB (NCBI 16S Microbial). In addition, multiple sequence alignments between ASAs were analyzed using the MAFFT (V7.475) program, while the phylogenic tree was analyzed using the FastTreeMP (V2.1.10) program. Furthermore, QIIME (V1.9) [45], with the abundance and taxonomy information of the above OTUs, was used for comparative analysis of various microbial clusters. The α-diversity information was obtained using the Shannon Index and verified by examining the Rarefaction curve and Chao1 values. The β-diversity was determined using the Weighted/Unweighted UniFrac distance, and the flexibility was visualized via PCoA [45]. Moreover, the MetaCyc metabolic pathway of fecal microbiota was predicted using PICRUSt2 (Phylogenetic Investigation of Communities by Reconstruction of Unobserved States, Huttenhower Lab, Boston, MA, USA). ASVs with an NSTI (Nearest sequenced taxon index) above 2 were excluded from PICRUSt2 analysis. The function of each microbiota was visualized using the ggplot (V3.3.2) program, where the dissimilarity between microbiota was represented after PCoA analyses based on the Bray–Curtis distance.

### 4.7. FMT Analysis

**Figure 12:**
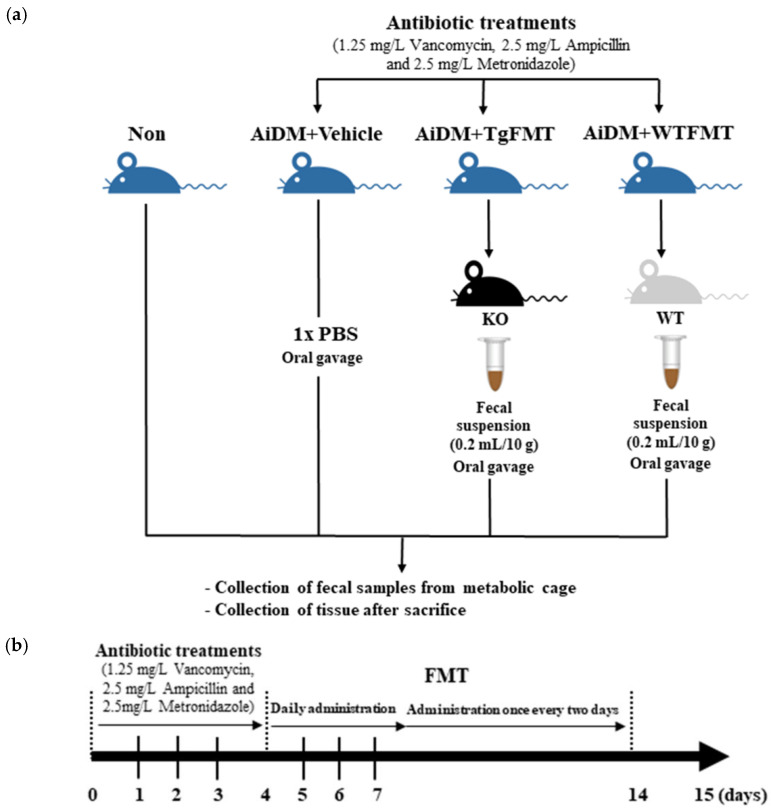
Experimental scheme for the FMT of the Tg2576 mice. (**a**) Treatment strategy. The WT and Tg2576 mice were divided into one of four groups: Non, AiDM+Vehicle, AiDM+TgFMT, and AiDM+WTFMT groups. The AiDM groups received the appropriate concentration of three antibiotics (vancomycin, ampicillin, and metronidazole) to remove intestinal bacteria. (**b**) Treatment schedule for FMT. After treatment with the three antibiotics for three days, the fecal suspensions of WT or Tg2576 mice were administrated orally into the AiDM-ICR mice seven times over two weeks. Abbreviations: WT, wild-type; Tg, Tg2576 mice; FMT, fecal microbiota transplantation; TgFMT, Tg2576 mice FMT; WTFMT, wild-type mice FMT; AiDM, antibiotics-induced depletion of microbiota.

FMT analysis was conducted as described elsewhere [46]. After collecting fresh fecal samples (10 g) from WT and Tg2576 mice, the fecal suspension solution was prepared through sequential procedures, such as suspension in a sterile 0.2 mL of 1× PBS solution, centrifugation, and filtration through a 0.45 µm syringe filter. They were stored at −70 °C in an Eppendorf tube until assayed. Eight-week-old ICR mice (*n* = 40) were assigned to either a Non-treated group (*n* = 10) or an antibiotics-induced depleted microbiota group (AiDM group, *n* = 30). To produce the AiDM model, a mixture of 1.25 mg/L vancomycin (1 g, BCworld Pharmaceutical Co., Ltd., Seoul, Republic of Korea), 2.5 mg/L ampicillin (5 g, Samyang Anipharm Co., Ltd., Seoul, Republic of Korea), and 2.5 mg/L metronidazole (1 mg, CJ Cheil Jedang Inc., Seoul, Republic of Korea) in a 1× PBS solution was administered daily to the ICR mice for three days by gavage. The Non-treated group received only 1× PBS alone under the same schedule. The AiDM mice (*n* = 30) were divided equally into the Vehicle-treated group (AiDM+Vehicle group, *n* = 10), Tg2576 fecal microbiota transplantation group (AiDM+TgFMT group, *n* = 10), or a WT fecal microbiota transplantation group (*n* = 10, AiDM+ WTFMT group). The fecal suspension from the WT mice (10 g feces in 0.2 mL of a 1× PBS solution) were transplanted daily to the WTFMT group mice by gavage for three days, while the fecal suspension from the Tg2576 mice (10 g feces in 0.2 mL of a 1× PBS solution) were transplanted daily to the TgFMT group mice using the same method. Subsequently, the same group was treated with these suspensions once every two days for 10 days. After the final treatment, the mice in the subset group were placed in metabolic cages for 24 h. At 10 a.m. on the 15th day, the total stools and urine were collected from the metabolic cage of each mouse, and the levels of these parameters were measured using the method suggested earlier. Subsequently, all mice in the subset group were euthanized with CO_2_ gas, after which the intestine tissues were harvested and stored at −70 °C in Eppendorf tubes until assayed (Figure 12).

### 4.8. Culture of Fecal-Derived Microbiota

The growth of microorganisms derived from the fecal samples of the WT and Tg2576 mice was measured using the viable cell count method. After collecting the feces samples from the mice of a subset group, they were suspended and serially diluted 1000–10,000 fold with sterile water. Each diluent was inoculated and spread on a nutrient agar plate comprised of peptone (0.5%), beef extract/yeast extract (0.3%), agar (1.5%), and NaCl (0.5%), and aerobically incubated at 30 °C for 24 h. The growth pattern of the bacteria colonies in the nutrient agar plate was observed on the view box.

### 4.9. Quantitative Real-Time PCR Analysis (RT-qPCR)

The relative quantities of AQP3, AQP8, 5-HT 2A, 5-HT 2B, 5-HT 3A, and 5-HT 3B mRNAs were determined with RT-qPCR, as described in a previous study [40]. After isolating the total RNA molecules from the frozen mid colon tissues, RT-qPCR was conducted with a cDNA template and 2× Power SYBR Green (Toyobo Co., Osaka, Japan) using the specific primers (Supplement Table S2). The fluorescence intensity, threshold values, and threshold cycle (Ct) were determined, as described elsewhere. The mRNA level of the specific gene was quantified relative to that of the β-actin, housekeeping gene, based on comparing the Cts at a constant fluorescence intensity and using the Livak and Schmittgen method [47].

### 4.10. Western Blotting Analysis

The total tissue homogenates were harvested from the mid colons of the mice using the Pro-Prep Protein Extraction Solution (Intron Biotechnology Inc., Seongnam, Republic of Korea) based on the manufacturer’s protocol. After collecting the total proteins, their concentrations were determined using a SMARTTM Bicinchoninic Acid Protein assay kit (Thermo Fisher Scientific Inc., Wilmington, MA, USA). The total 30 μg proteins were separated on 4–20% sodium dodecyl sulfate–polyacrylamide gel electrophoresis (SDS-PAGE) for 3 h, and transferred to nitrocellulose membranes for 2 h at 40 V. Each membrane was incubated with the following primary antibodies overnight at 4 °C: anti-C-kit (DAKO, Kyoto, Japan), anti-NSE (Abcam Com., Cambridge, UK), anti-PGP9.5 (Abcam Com.), anti-cleaved Cas-3 (Abcam Com.), anti-mAChR M2 (Alomone Labs, Jerusalem, Israel), anti-mAChR M3 (Alomone Labs), anti-Gα (Abcam Com.), and anti-actin (Sigma-Aldrich Co.). After removing the unbound antibodies, the membrane was incubated with 1:1000 diluted horseradish peroxidase–conjugated goat anti-rabbit IgG (Zymed Laboratories, South San Francisco, CA, USA) for 2 h at room temperature. Subsequently, all membrane blots were developed using a Chemiluminescence Reagent Plus kit (Pfizer Inc., Gladstone, NJ, USA). Luminescence signals of specific proteins were acquired on a digital camera (1.92 MP resolution) using the FluorChem^®^ FC2 imaging system (Alpha Innotech Corporation, San Leandro, CA, USA). Their densities were semi-quantified using the AlphaView Program, version 3.2.2 (Cell Biosciences Inc., Santa Clara, CA, USA).

### 4.11. Measurement of 5-HT Concentrations

The 5-HT concentration was quantified using ELISA kits (ImmuSmol, Pessac, France), according to the manufacturer’s instructions. Firstly, the mid colon tissues (20 mg) of WT and Tg2576 mice were homogenized in ice-cold 1× PBS (pH 7.2–7.4) using a glass homogenizer (Sigma-Aldrich Co.). The tissue homogenates and diluted standards lysates were acylated for 30 min at room temperature on a shaker, and they were mixed with serotonin antiserum samples for 15–20 h at 2–8 °C. After washing, enzyme conjugate was added to all wells for 30 min at room temperature. The substrate reagent was added and incubated for 30 min at room temperature, and this reaction was quenched by adding the stop solution. Finally, the absorbance of the reaction mixture was read at 450 nm using the VersaMax Plate Reader (Molecular Devices, Sunnyvale, CA, USA).

### 4.12. Statistical Analysis

The data are presented as mean ± standard deviation (SD) values. The statistical significance was evaluated by one-way analysis of variance (ANOVA) (SPSS for Windows, Release 10.10, Standard Version, Chicago, IL, USA) followed by a Tukey’s post hoc t-test for multiple comparisons. A *p* value < 0.05 was considered significant.

## 5. Conclusions

This study examined the correlation between dysbiosis on the fecal microbiota and constipation in Tg2576 mice during the pathogenesis of AD. These results provide scientific evidence that the neuropathological constipation of Tg2576 mice is associated with the dysregulation of Bacteroidetes, Firmicutes, and Proteobacteria in the fecal microbiota. Furthermore, these results verify these alterations in the fecal microbiota in Tg2576 mice through an FMT experiment using the AiDM model. Furthermore, the microbial populations identified in this study are potential therapeutic targets for treating constipation associated with neurodegenerative diseases, such as AD and PD.

## Data Availability

All the data that support the findings of this study are available on request from the corresponding author.

## Supplementary Materials

The following supporting information can be downloaded at: https://www.mdpi.com/article/10.3390/ijms232314928/s1.
