# Supplementary material for: Dysbiosis of Fecal Microbiota in Tg2576 Mice for Alzheimer’s Disease during Pathological Constipation
Source: Int J Mol Sci. 2022 Nov 29;23(23):14928. doi: 10.3390/ijms232314928 (PMC9736912; doi:10.3390/ijms232314928)
Supplement: Supplementary file 1 [file ijms-23-14928-s001.zip › ijms-1819797-supplementary.pdf]

Supplement Table S1. Universal primer pair with the illumina adapter overhang

Gene	Primer (5′→3′)
V3-Forward	TCGTC GGCAG CGTCA GATGT GTATA AGAGA CAGCC TACGG GNGGC WGCAG
V4-Reverse	GTCTC GTGGG CTCGG AGATG TGTAT AAGAG ACAGG ACTAC HVGGG TATCT AATCC

Supplement Table S2. Sequence of primers used for RT-PCR analysis

Gene	Forward primer (5′→3′)	Reverse primer (5′→3′)
AQP3	GGTGG TCCTG GTCAT TGGAA	AGTCA CGGGC AGGGT TGA
AQP8	GTAGTATGGACCTACGTGAGAT CAAGG	AGAACCTTTCCTCTGGACTCACC ACC
5-HT 2AR	CCGGG AGCCT CTTGA TACAG	AGCCC CTCTC AAAGT CACAC A
5-HT 2BR	GCAGA TTTGC TGGTT GGATT G	GGCCA TATAG CCTCA AACAT GAT
5-HT 3AR	CTGAG GCCCT CCCAC ATCT	GGAAA GGAAC AAGGC CAACA
5-HT 3BR	TGCCG AGGAG TCTAG ATTGT ACCT	ACCCG ATGCT CCTGA TGGA
β-actin	ACGGC CAGGT CATCA CTATT G	CAAGA AGGAA GGCTG GAAAA GA
